# Silk peptide-hyaluronic acid based nanogels for the enhancement of the topical administration of curcumin

**DOI:** 10.3389/fchem.2022.1028372

**Published:** 2022-09-19

**Authors:** Jiangxiu Niu, Ming Yuan, Yao Liu, Liye Wang, Zigui Tang, Yihan Wang, Yueheng Qi, Yansong Zhang, Huiyuan Ya, Yanli Fan

**Affiliations:** ^1^ College of Food and Drug, Henan Functional Cosmetics Engineering and Technology Research Center, Luoyang Normal University, Luoyang, Henan, China; ^2^ Department of Pharmacy, Henan Medical College, Zhengzhou, China

**Keywords:** silk peptide, hyaluronic acid, curcumin, nanogels, topical administration

## Abstract

The present study focused on the development of Cur-loaded SOHA nanogels (Cur-SHNGs) to enhance the topical administration of Cur. The physiochemical properties of Cur-SHNGs were characterized. Results showed that the morphology of the Cur-SHNGs was spherical, the average size was 171.37 nm with a zeta potential of −13.23 mV. Skin permeation experiments were carried out using the diffusion cell systems. It was found that the skin retention of Cur-SHNGs was significantly improved since it showed the best retention value (0.66 ± 0.17 μg/cm^2^). In addition, the hematoxylin and eosin staining showed that the Cur-SHNGs improved transdermal drug delivery by altering the skin microstructure. Fluorescence imaging indicated that Cur-SHNGs could effectively deliver the drug to the deeper layers of the skin. Additionally, Cur-SHNGs showed significant analgesic and anti-inflammatory activity with no skin irritation. Taken together, Cur-SHNGs could be effectively used for the topical delivery of therapeutic drugs.

## 1 Introduction

Topical transdermal drug delivery is one of the important methods for the treatment of local diseases (such as contact dermatitis, psoriasis, etc.) (Dasht Bozorg et al., 2022). For some locally effective drugs, compared with oral and injection routes, topical transdermal administration can reduce the entry of drugs into the blood circulation, thereby reducing systemic adverse reactions (Hanna et al., 2019). Besides, the slow release of topical drugs into the skin tissue can prolong the efficacy time, reduce the number of doses, provide patients with greater comfort (Jiang et al., 2018). Although topical transdermal administration has many advantages, the stratum corneum of the skin limits the transdermal penetration of external agents, resulting in drug waste and poor clinical efficacy (Truong et al., 2022; Uner et al., 2022). Therefore, for topical drug formulations, the primary goal of formulation development is to maximize drug penetration through the skin while maximizing drug retention in the skin.

To improve the transdermal penetration and the retention of drugs in the skin, various nanocarriers (such as solid lipid nanoparticles, ethosomes, and emulsions) have been developed to improve topical transdermal delivery (Ilic et al., 2021; El-Hashemy, 2022; Niu et al., 2022). Among various types of nanocarriers, nanogels have attracted increasing attention due to their unique advantages, including great colloidal stability, tunable size, large surface area for biological coupling, and porous structure for loading large amounts of drugs (Sivaram et al., 2015; Liu et al., 2021a). In addition, due to the unique structure, nanogels can bind a large amount of water to moisturize and enhance the hydration of the stratum corneum, which is conducive to its role in the field of transdermal drug delivery (Kim et al., 2021). A variety of natural and synthetic polymer nanogels have been prepared and used as transdermal drug delivery vehicles to date (Uk Son et al., 2020; Wang et al., 2022).

Hyaluronic acid (HA) is a linear polysaccharide consisting of repeating units of D-glucuronic acid and N-acetyl-D-glucosamine (Jiang et al., 2022). As a natural polymer, HA has been widely studied in drug delivery due to its outstanding biological function (Yu et al., 2021b). It is well known that HA can overcome the skin barrier by hydrating the stratum corneum, and effectively delivering drugs to the deeper layers of the skin (Chen et al., 2020). In addition, some studies have proved the applicability of HA based nanocarriers in enhancing topical drug delivery due to the interaction between HA based nanocarriers and keratin components in skin (Liu et al., 2021b; Kim et al., 2021; Sharma et al., 2022). However, HA is hydrophilic and is not suitable as a carrier for hydrophobic drugs. It has been reported that intramolecular and/or intermolecular hydrophobic interactions can self-aggregate in aqueous solutions to form nanogels when HA is hydrophobized, and the hydrophobic regions inside the nanogels are beneficial for the loading and topical delivery of hydrophobic drugs (Son et al., 2017).

In recent years, silk fibroin has attracted great interest due to its biological adhesion and its application in drug stabilization, and is currently used in various drug delivery systems such as nanoparticles, hydrogels and nanofibers (Chen et al., 2015; Sakunpongpitiporn et al., 2022; Selvaraj et al., 2022). Silk peptide is produced by hydrolysis of silk fibroin. It is non-toxic to human skin and has high affinity, water retention and anti-inflammatory properties (Eom et al., 2020).

Curcumin (Cur), a natural polyphenol extracted from the rhizome of turmeric with various pharmacological effects. The topical application of Cur to the skin has long been of great interest in anti-oxidation, anti-inflammatory and light protection (Shi et al., 2020). However, chemical instability, low water solubility, and poor skin penetration are major obstacles to their application as topical therapeutics (Granata et al., 2020). Therefore, finding novel formulations aimed at overcoming these limitations and enhancing the topical pharmacological properties of Cur remains a challenge. Currently, Cur-loaded nanoparticles (such as solid lipid nanoparticles, liposomes, emulsions, micelles, etc.) seem to be a very promising formulation method to effectively improve the topical efficacy of Cur (Vater et al., 2020; Yu et al., 2021a; Zhou et al., 2021; Prabhu et al., 2022).

**FIGURE 1:**
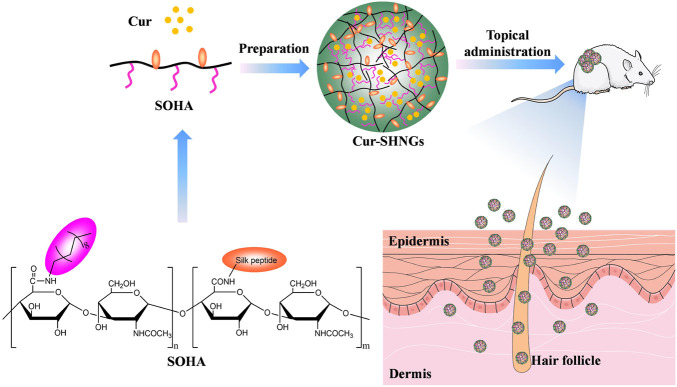
Schematic illustration of the Cur-loaded nanogels (Cur-SHNGs) based on SOHA conjugate for the enhancement of the topical administration of Cur.

In this study, silk peptide conjugated OHA (SOHA) was successfully synthesized on the basis of octadecylamine conjugated hyaluronic acid (OHA), and Cur-loaded SOHA nanogels (Cur-SHNGs) were developed to enhance the topical administration of Cur for better treatment of localized diseases (Figure 1). The physicochemical properties of micelles were characterized in term of particle size, zeta potential, morphology, stability, and *in vitro* release. In addition, the *in vitro* transdermal penetration and skin retention, effect of the preparation on skin structure, intradermal drug distribution, *in vivo* analgesic and anti-inflammatory activities were also investigated. Finally, biocompatibility assessments were performed using *in vivo* skin irritation test.

## 2 Materials and methods

### 2.1 Materials

Hyaluronic acid (HA, molecular weight <10 *k*Da) was purchased from Freda Biochem Co., Ltd. (Shandong, China). Silk peptide (Protein hydrolyzates, molecular weight is between 500 and 1,000 Da) was purchased from Shanghai McLean Biochemical Technology Co., Ltd. (Shanghai, China). Octadecylamine was purchased from Aladdin reagent Co., Ltd. (Shanghai, China). (3-dimethylaminopropyl)-3-ethylcarbodiimide hydrochloride (EDC.HCL) and N-hydroxysuccinimide (NHS) were purchased from Nanjing dulai Biotechnology Co., Ltd. (Nanjing, China). Curcumin was purchased from Ivy Biotechnology Co. Ltd. (Xian, China). Haematoxylin and Eosin staining were purchased from Sigma-Aldrich (Shanghai, China). All other reagents were analytical grade preparation.

### 2.2 Animals

Female SD rats (180–220 g) and female ICR mice (18–22 g) were used as the experimental animals. Animals were raised in the animal care facility of the Pharmacology Laboratory of Luoyang Normal University. Animal room was kept at 22°C ± 3°C and under a 12 h light cycle, with free access food and water. All animal feeding and experimental procedures were guided by the guidelines of Henan Provincial Experimental Animal Management Committee and approved by the Animal Ethics Committee of Luoyang Normal University.

### 2.3 Synthesis of SOHA conjugate

Octadecylamine conjugated hyaluronic acid (OHA) was formed by covalently combining the −NH_2_ group in octadecylamine with the −COOH group of HA through an amide reaction using EDC and NHS as catalysts (Son et al., 2017). Briefly, hyaluronic acid solution (1%, w/v) was prepared in deionized water by naturally swell. A double molar excess of EDC and NHS was added to activate the −COOH groups in HA, followed by the addition of octadecylamine (dissolved in DMF). The molar ratio of HA subunit: octadecylamine was 1:1. The reaction was first run at 60°C for 5 h, and then at room temperature for 24 h. After the reaction was stopped, the mixture was dialyzed in ethanol/deionized water (V/V, 7:3, 5:5, 3:7) for 2 days and further freeze-dried to obtain dry purified OHA conjugate. The structure of OHA was characterized by ^1^H NMR using an instrument (AVANCE500, Bruker, Germany).

Silk peptide (440 mg) was dissolved in 5 ml of DMF. OHA conjugate (100 mg) was fully dissolved in 10 ml of dimethylformamide (DMF), followed by addition of EDC (100 mg) and NHS (60 mg), the mixture was stirred at room temperature for 6 h and then the silk peptide solution was slowly dropped into the OHA activated solution. The reaction was run at room temperature for 24 h under stirring. The ultimate reaction solution was dialyzed against an excess amount of deionized water for 48 h, and then the solution was filtered to remove impurities through a 0.45 μm millipore filter. Finally, silk peptide conjugated OHA (SOHA) was collected by freeze-drying. The structure of SOHA was characterized by ^1^H NMR.

### 2.4 Preparation and characterization of Cur-SHNGs

#### 2.4.1 Preparation of Cur-SHNGs

Cur-SHNGs were prepared by dialysis-sonication method. Briefly, 160 mg of SOHA and 8 mg of Cur were mixed in 10 ml of deionized water and 3 ml of DMSO, respectively. The mixture was dialyzed against deionized water in the dark for 48 h using a dialysis bag (MWCO 3.5 kDa) with stirring. After dialysis, the mixture was subjected to ultrasonic treatment for 20 min. Subsequently, the sample was freeze-dried with a freeze dryer (SCIENTZ-18ND, Ningbo Xinzhi Biotechnology Co., Ltd., China) to obtain Cur-SHNGs powder. Nanogels consisting of Cur and formulated by OHA (Cur-HNGs) were prepared in the same way.

#### 2.4.2 Quantitation of Cur using HPLC

Cur in the formulation was quantified using a high performance liquid chromatography (HPLC) system (U-3000, Thermo, United States) equipped with a UV detector and a reversed-phase WondaSil C18 column (5 mm, 200 mm × 4.6 mm). The mobile phase was composed of acetonitrile and 0.5% phosphoric acid (58:42, V/V), and the flow rate was 1.0 ml/min. The wavelength of the UV detector and the temperature of the column oven were set to 423 nm and 30°C, respectively. The injection volume was 20 μl. The Cur content (%) in the nanogels was calculated according to following formula: Cur content (%) = (mass of Cur loaded in nanogels/mass of Cur loaded nanogels) × 100%.

#### 2.4.3 Measurement of particle size and zeta potential

The particle size and zeta potential of the nanogels were determined using a dynamic light scattering instrument (Zetasizer ZS90, Malvern Instruments, United Kingdom). Samples were diluted to 3.0 mg/ml with deionized water prior to determination. Each sample was placed in a zeta potential cell and the zeta potential of the particles was measured at 25°C. The results were the average of three measurements for each sample.

#### 2.4.4 Morphological observations of Cur-SHNGs

To observe the morphology of the Cur-SHNGs particles, scanning electron microscope (SEM) was performed using (Sigma 500, ZEISS, Germany). The Cur-SHNGs were diluted to 2 mg/ml, the diluted sample was dropped on the surface of the silicon wafer and kept at room temperature until completely dry. A small amount of freeze-dried Cur-SHNGs was placed on the conductive glue. The samples were coated with gold before observing. The SEM images of dispersed and lyophilized Cur-SHNGs were scanned and recorded, respectively.

#### 2.4.5 Stability of Cur-SHNGs

To investigate the stability of Cur-SHNGs, lyophilized Cur-SHNGs powder was re-dispersed in deionized water and stored at 4°C in the dark for 0, 7, 14, and 21 days. At predetermined time points, the content of Cur was determined by HPLC system, the particle size and zeta potential were measured by dynamic light scattering instrument.

### 2.5 *In vitro* release

*In vitro* release studies were conducted in triplicate in phosphate buffered saline (PBS, pH 7.4) containing ethanol (40%, v/v). 2 ml of Cur solution, Cur-HNGs or Cur-SHNGs (0.5 mg/ml) were transferred into dialysis bags (MWCO 3500). Subsequently, the bags were sealed and immersed in 20 ml of release medium. The release medium was continuously stirred at 300 rpm at 37°C. At preset sampling time points (0.5, 1, 2, 4, 8, 12, 24, and 48 h), 2 ml samples were withdrawn and an equal amount of corresponding fresh release medium was placed back. The concentration of Cur in the samples was measured using a fluorescence spectrophotometer (Ex = 442 nm, Em = 475 nm) (F-7000, Hitachi High-Tech, Japan). The cumulative release of Cur was calculated by the following formula: (Liu et al., 2021a)

$$Cumulative\;release\;of\;Cur\;\left(\%\right)=\frac{V_{i}\sum_{1}^{n-1}C_{i}+V_{0\;}C_{n}}{m}\times 100\%$$

Where: V_i_ is the volume of samples (ml), V_0_ is the total volume of the release medium (ml), C_i_ is the drug concentration of the samples (mg/ml), m is the initial amount of the Cur in the dialysis bags (mg), and n is the number of samples (n > 0).

### 2.6 *In vitro* skin permeation studies

#### 2.6.1 Preparation of rat skin

After the abdominal hair of the rats was shaved with an electric shaver, the animals were sacrificed by ether inhalation anesthesia, the abdominal skin was excised, adipose tissue and adhesions were removed, and then the skin was hydrated with physiological saline and stored at 4°C for use (Daryab et al., 2022).

#### 2.6.2 *In vitro* permeation studies in rat skin

*In vitro* skin penetration study of Cur from Cur solutions, Cur-HNGs and Cur-SHNGs on rat skin was implemented using the transdermal diffusion cell systems (Ali et al., 2021; Sudhakar et al., 2021). The skin membrane was fixed between the donor chamber and receiving chamber of the diffusion cell, with the epidermis facing the donor chamber and the dermis facing the receiving chamber. The receiving chamber was filled with phosphate buffered saline (PBS, pH 7.4) containing ethanol (40%, v/v). Subsequently, 0.3 ml of Cur solution, Cur-HNGs or Cur-SHNGs (equivalent to 0.15 mg Cur) was added to the donor chamber. The glass tube was suspended in a water bath at 37°C ± 0.5°C and agitated at 300 rpm. Samples of 1.0 ml were taken from the receiver chamber at predetermined time intervals up to 24 h and immediately replenished with an equal amount of receiving solution. The amount of Cur was quantified using HPLC as described above.

After the penetration experiment was completed, the skin samples were removed from the diffusion cell and rinsed with deionized water to remove residual formulation from the surface of the skin. The skin was then cut into pieces and subjected to intermittent sonication in 2 ml of methanol for 120 min to extract the drug in the skin. The Cur retained in the skin was quantitatively analyzed by HPLC.

### 2.7 Hematoxylin and eosin staining

The back hair of the mice was depilated 1 day before the experiment, and then Cur solutions, Cur-HNGs and Cur-SHNGs (containing 0.15 mg of Cur) were applied in the depilation area. 6 h after application of the formulations, the mice were killed and the skin was immediately collected. After washing with physiological saline, the skin was placed in a 4% paraformaldehyde solution at 4°C for fixation. The fixed skin was embedded in paraffin, and serial longitudinal sections were obtained with a thickness of 5 μm using a microtome (LEICA RM2235, Nussloch, Germany). Photographs of paraffin sections were obtained using a digital slide scanner (3DHISTECH, Ltd.), and the effect of formulations on skin microstructure was analyzed using the matching analysis software of CaseViewer 2.3.

### 2.8 Fluorescence imaging

Different Cur formulation (Cur solution, Cur-HNGs, and Cur-SHNGs) was respectively administered to the skin of living mice as described in section of “Hematoxylin and eosin staining.” After 1 and 6 h of administration, the skin samples were excised and thoroughly washed with physiological saline. Subsequently, the treated skin samples were cut longitudinally using a freezing slicer (Leica CM 1950, Germany). The skin sections were nuclear counterstained with DAPI, fluorescent images were acquired using a digital slide scanner (3DHISTECH, Ltd.).

### 2.9 *In vivo* hot plate test in mice

*In vivo* analgesic activity studies in mice were performed using the hot plate method (Abdallah et al., 2021). Briefly, each mouse was individually placed on the hot plate of a smart hot plate apparatus (YLS-6B, Jinan, China) maintained at 55°C ± 1°C. The time elapsed from the onset of mouse exposure to the hot plate to eliciting a response (including paw licking, paw withdrawal, limb lift, or jumping) was recorded as the latency (Muangnoi et al., 2018). Female mice with a basal latency of 5–30 s were selected for testing. The mice were divided into four groups with 10 mice in each group. After the determination of the basal latency, the hot plate latencies were recorded at different time points (30, 60, and 120 min) post the administration of Cur solution, Cur-HNGs or Cur-SHNGs at a dose of 15 mg/kg. The cutoff time was set at 60 s.

### 2.10 Dimethyl benzene induced mice ear edema test

The mice were randomly divided into four groups with 10 mice in each group. Cur solution, Cur-HNGs or Cur-SHNGs were uniformly applied to the two sides of the right ear in each group at the dose of 0.4 mg/kg. After treatment for 1 h, 30 μl of dimethyl benzene was applied topically on both sides of the right ear (15 μl on each side) to induce ear edema. The mice were humanely killed after treatment with dimethyl benzene for 30 min, and then the same area of the left and right ears of the mice were carefully cut off with a puncher, the ears (8 mm in diameter) of all mice were harvested and weighed accurately. The ear edema and ear swelling inhibition rate were calculated.

### 2.11 Acute dermal irritation test

The hairs on both sides of the spine (approximately 3 cm × 3 cm) were shaved 24 h before the experiment. The mice were divided into four groups of six mice each. Then 0.5 ml of Cur solution, Cur-HNGs or Cur-SHNGs was administered on depilated dorsal surface of the mice, the formulation was removed after 4 h of exposure, and any visible changes in the skin (such as erythema, edema, etc.) were observed at 1, 4, 24, 48, and 72 h.

### 2.12 Statistical analysis

Results were expressed as mean ± standard deviations (SD) of triplicate experiments. The statistical differences among groups were analyzed using Student’s *t*-test, and *p* < 0.05 was considered significant.

## 3 Results and discussions

### 3.1 Synthesis and characterizations of SOHA conjugate

**FIGURE 2:**
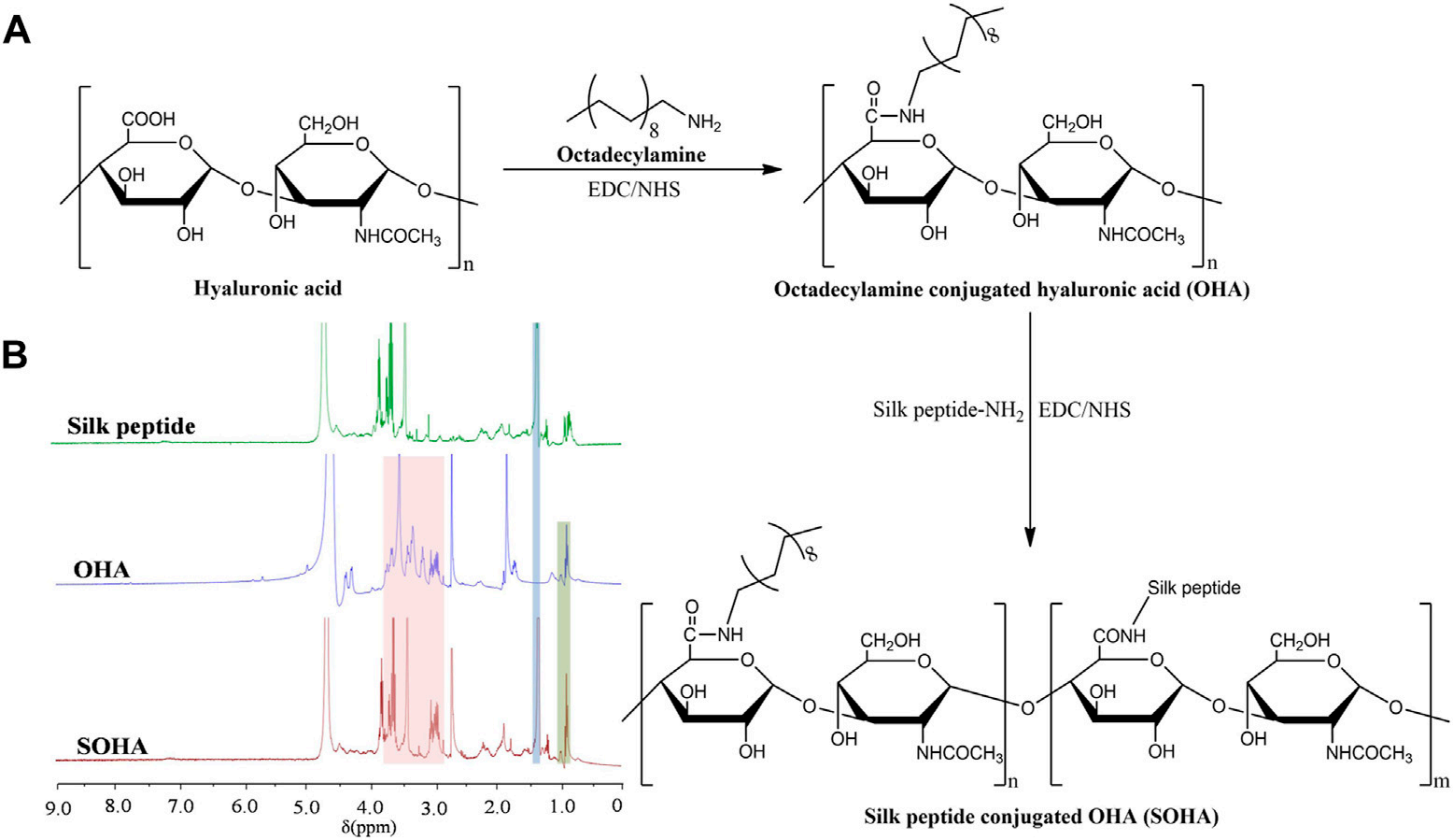
**(A)** Synthetic scheme of SOHA. **(B)** ^1^H NMR spectra of silk peptide, OHA and SOHA.

The synthetic scheme of SOHA conjugate is shown in Figure 2A. Under the catalysis of EDC and NHS, a new conjugate SOHA was synthesized through the amide reaction between the −COOH groups of HA and the −NH_2_ groups of octadecylamine and silk peptide. Figure 2B showed the ^1^H NMR spectra of silk peptide, OHA, and SOHA. The characteristic peak of SOHA at 0.95 ppm was the chemical shift corresponding to protons of the −CH_3_− group in octadecylamine (Yuan et al., 2022), the peak at 1.24 ppm was attributed to the chemical shifts of the −CH−NH_2_CH_3_ proton in alanine of silk peptide (Zainuddin et al., 2008), and peaks ranging from 2.94 to 3.85 ppm were the chemical shift corresponding to protons of the glycosides in HA (Yuan et al., 2022). The ^1^H-NMR spectrum indicating that SOHA conjugate was successfully synthesized. The substitution degree (DS) of octadecylamine and silk peptide was 14.82% ± 0.27% and 9.46% ± 0.15% in SOHA conjugate, respectively.

#### 3.2 Preparation and characterization of the Cur-SHNGs

### 3.2.1 Preparation of Cur-SHNGs

Hyaluronic acid is a water-soluble polymer that could form gels with a network structure (Zhang et al., 2019). It has been reported that when HA polymers are hydrophobized by hydrophobic groups, self-aggregating nanogels could be formed (Zoratto et al., 2021). In this study, Cur-loaded SOHA nanogels (Cur-SHNGs) were formulated by dialysis-sonication method. This method could not only easily embed a large amount of highly lipophilic drugs, but also form nanogels with small and uniform particle size. When the weight ratio of Cur to SOHA was 1:20, the content of Cur was 4.26%, which showed a satisfactory drug content and no precipitation was found during preparation.

### 3.2.2 Particle size and zeta potential

**FIGURE 3:**
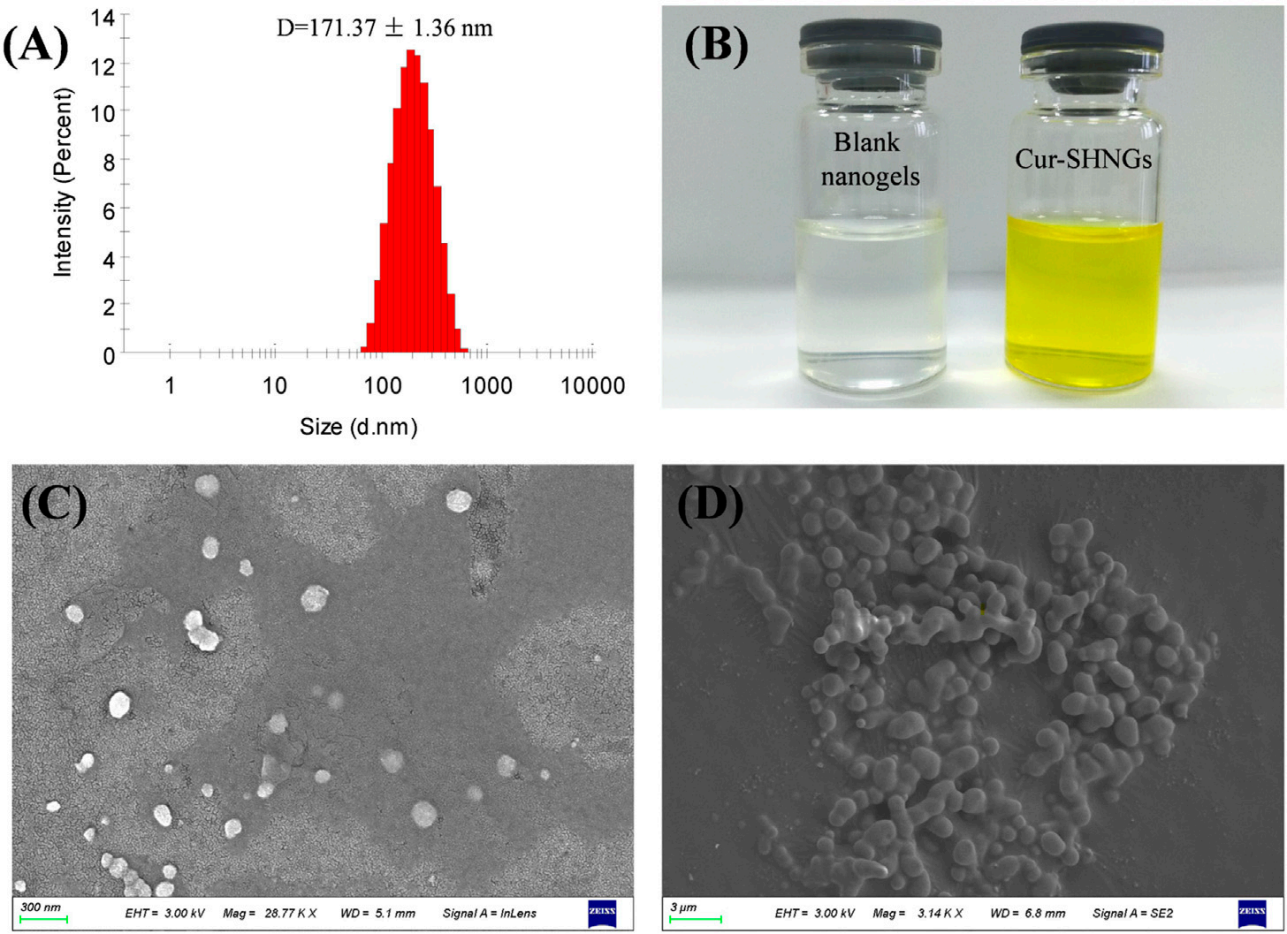
Characterization of Cur-SHNGs. **(A)** Particle size distribution of Cur-SHNGs. **(B)** Appearance of blank nanogels and Cur-SHNGs. **(C)** SEM image of Cur-SHNGs dispersion.**(D)** SEM image of lyophilized Cur-SHNGs powder.

In skin application, the particle size of the formulation plays an important role. Usually, when the particle size of the formulation is less than 300 nm, it is easy to penetrate the stratum corneum and form a drug reservoir in the skin. However, when the particle size is less than 100 nm, it is easier to enter the blood circulation through the dermis (Verma et al., 2003). The particle size of blank nanogels was 163.16 ± 2.31 nm with a PDI of 0.15 ± 0.02. After loading Cur, Cur-SHNGs presented a slightly increased particle size of 171.37 ± 1.36 nm with a PDI of 0.23 ± 0.01. Only one peak was observed in the diagram of particle size distribution of the Cur-SHNGs (Figure 3A), which indicated the uniform particle size distribution of the formulation. The loading of Cur did not significantly change the particle size of the nanogels. The particle size of Cur-SHNGs is between 100 and 300 nm. Therefore, Cur-SHNGs might enter deeper skin through the stratum corneum and form a drug reservoir in the skin. The average size of Cur-HNGs was around 165 nm, so the conjugation of silk peptide did not obviously change the size.

The zeta potential of nanoparticles might be one of the important factors affecting their stability and skin deposition (Kahraman et al., 2018). The zeta potentials of blank nanogels, Cur-HNGs and Cur-SHNGs were −12.90 ± 0.21, −27.08 ± 1.34, and −13.23 ± 0.90 mV, respectively. Compared with Cur-HNGs, the negative charge of Cur-SHNGs was weakened, which might be due to the binding of silk protein peptides. It was found that nanoparticle systems with a zeta potential greater than ±30 mV can produce good stability of the suspension, while less than ±15 mV might lead to the problem of aggregation and sedimentation (Deng et al., 2021). Nevertheless, the stability experiments showed that the stability of the Cur-SHNGs was not affected by the surface zeta potential (discussed in section of “Stability of Cur-SHNGs”). Since skin cells are negatively charged, lower repulsion between Cur-SHNGs with less negative charge and skin cells might result in better cell adsorption and intradermal drug deposition (Zhang et al., 2020).

### 3.2.3 Morphology of Cur-SHNGs

The prepared blank nanogels were uniform and transparent in appearance, while the formulated Cur-SHNGs were yellow and homogeneous (Figure 3B). As shown in Figure 3C, the Cur-SHNGs dispersion appeared as a single nanoparticle with spherical shape. The lyophilized powder of Cur-SHNGs also presented a spherical shape with a smooth surface (Figure 3D).

### 3.2.4 Stability of Cur-SHNGs

**FIGURE 4:**
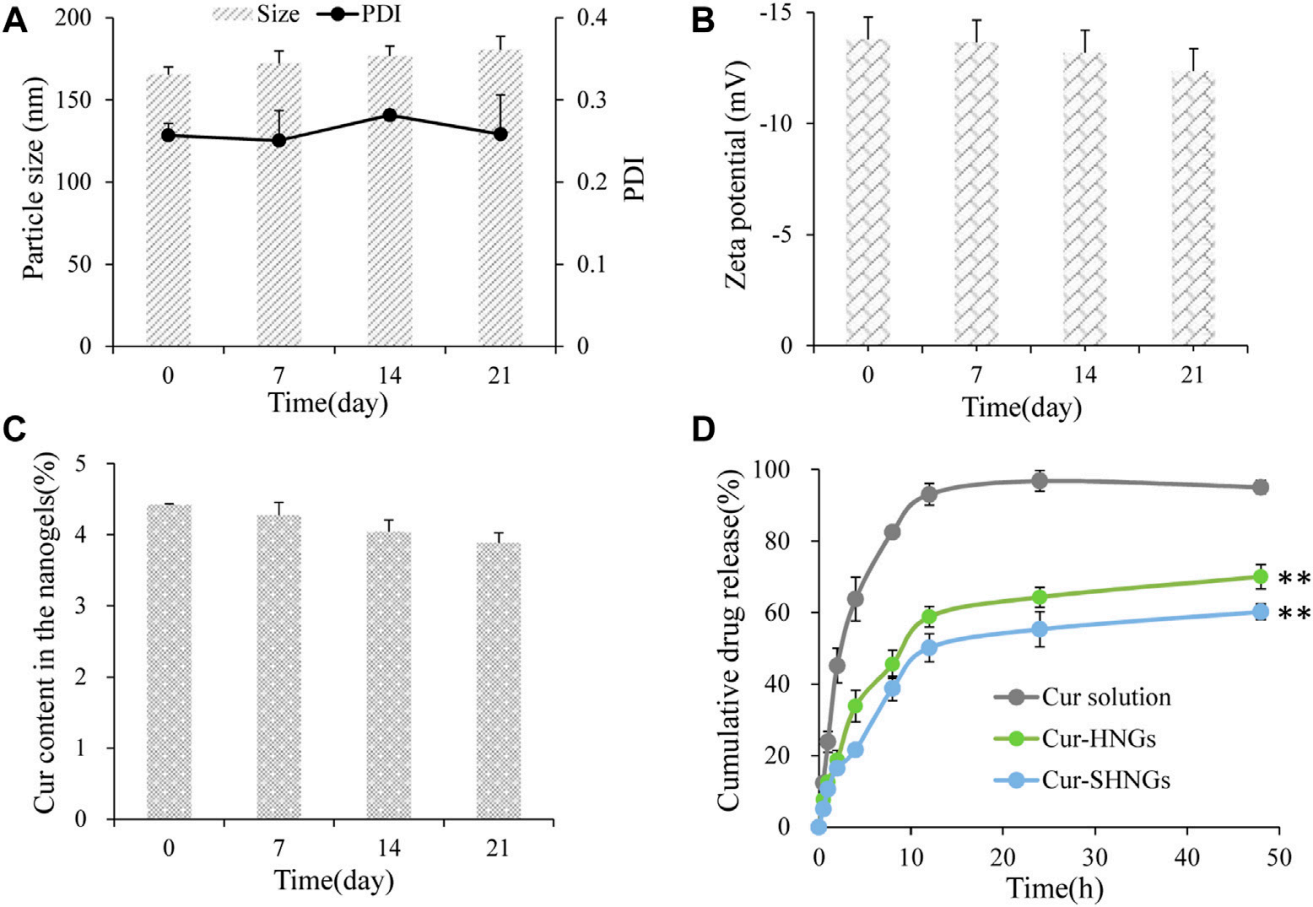
The storage stability of Cur-SHNGs at 4°C by **(A)** particle size and PDI, **(B)** zeta potential and **(C)** Cur content in the nanogels. **(D)** The *in vitro* release of Cur from Cur solution, Cur-HNGs, and Cur-SHNGs in PBS (pH 7.4) containing ethanol (40%, v/v) (results were presented as mean ± SD, *n* = 3; **p < 0.01 compared with the Cur solution).

After 21 days of storage in the dark at 4°C, the particle size, zeta potential value and Cur content of Cur-SHNGs were not statistically different from those observed at 0 days of storage, indicating that the gel network formed by Cur-SHNGs could protect Cur from degradation and oxidation, and effectively improve the physicochemical stability of the formulation Figures 4A–C.

### 3.3 *In vitro* release

The *in vitro* release curves were shown in Figure 4D. It was obvious that almost 95.06% ± 1.87% of the Cur was released from the Cur solution within 48 h, while the cumulative release of the Cur-HNGs and Cur-SHNGs was only 70.05% ± 3.39% and 60.23% ± 2.18%, respectively. The Cur-HNGs and Cur-SHNGs showed a significantly lower cumulative release compared to that of Cur solution at 48 h (*p* < 0.01). The slower release rate of Cur loaded nanogels might be attributed to the unique grid structure of the nanogels formed by the entanglement of OHA or SOHA chains, which affected the drug diffusion and slowed down drug release. This might be beneficial for the formulation to act as an intradermal drug depot and to release the drug continuously in the skin, rather than releasing the drug before the nanogels penetrate into the skin.

### 3.4 *In vitro* skin penetration and retention

**FIGURE 5:**
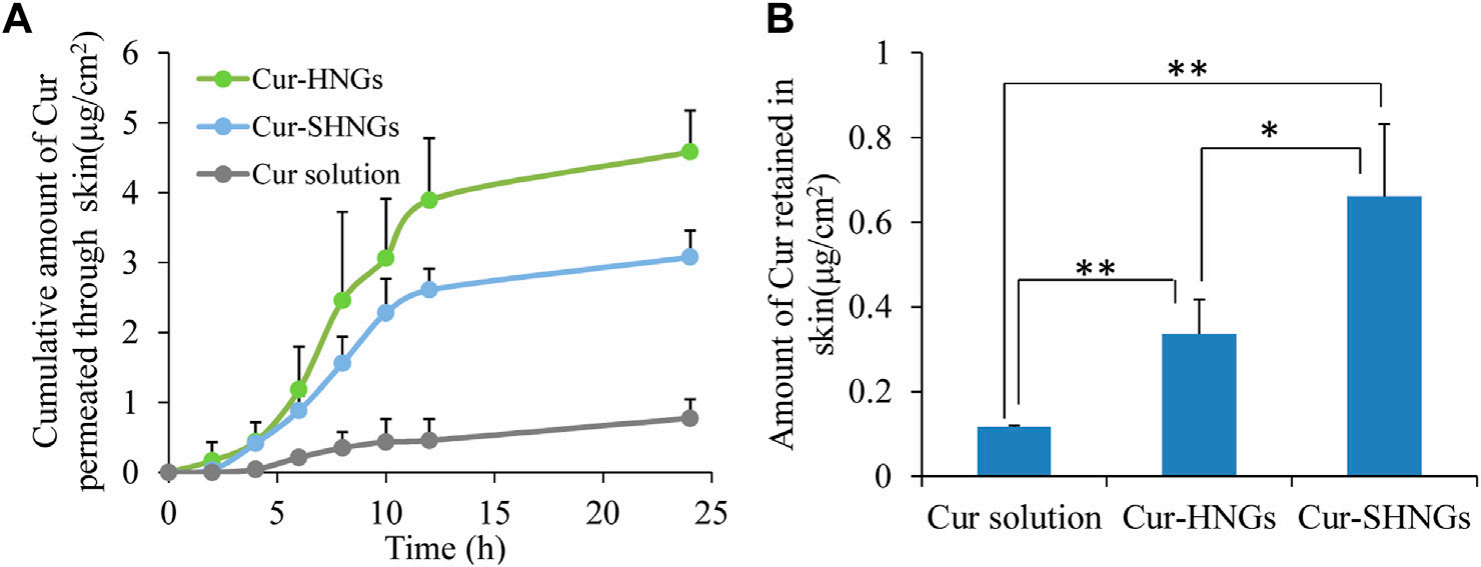
*In vitro* skin permeation studies of Cur solution, Cur-HNGs and Cur-SHNGs in rat skin after 24 h of topical administration. **(A)** The *in vitro* skin cumulative permeation of Cur through the skin. **(B)** Cur retention in the skin (results were presented as mean ± SD, *n* = 3; *p < 0.05, **p < 0.01).

Figure 5A showed the cumulative amount of Cur permeated through the unit area of abdominal rat skin from the Cur solution, Cur-HNGs and Cur-SHNGs. At each time point, the cumulative permeability of Cur-HNGs and Cur-SHNGs was much higher than that of Cur solution, no lag phase was observed for the Cur-HNGs and Cur-SHNGs, and Cur could be detected in the receiving chambers at the first time point of 2 h, indicated that the nanogels could quickly cross the stratum corneum and penetrate through the skin. While, only a small amount of drugs could be detected in the receiving chambers at 4 h for Cur solution. After 24 h of percutaneous penetration, the cumulative amount of Cur permeated through the skin from the Cur solution, Cur-HNGs and Cur-SHNGs was 0.77 ± 0.27, 4.58 ± 0.59, and 3.08 ± 0.38 μg/cm^2^, respectively. It was obvious that Cur permeated through rat skin from Cur-HNGs and Cur-SHNGs was significantly higher than that of Cur solution (*p* < 0.01). The small particle size of the formulated nanogels provided a large surface area that promotes the transdermal penetration of drugs (Elmataeeshy et al., 2018). In addition, it has been reported that HA has an unusually strong ability to absorb water and can bind 1,000 times its own volume of water (Zhu et al., 2020), so it can greatly hydrate the stratum corneum, thereby causing swelling of keratinocytes and reducing the compactness of the stratum corneum structure, ultimately increasing permeation efficiency of the drug (Zhu et al., 2020). The efficacy of topical therapeutic drugs depends on the amount of drugs in the skin, which is related to the ability of drugs to penetrate the stratum corneum into the skin tissue. Cur loaded nanogels had a stronger percutaneous permeability than that of the Cur solution, indicated that more Cur would be retained in skin, which is beneficial to the treatment of local diseases (Zheng et al., 2016; Faisal et al., 2018). The cumulative permeation of Cur through the skin at 24 h was found to be significantly reduced in the case of Cur-SHNGs compared to Cur-HNGs (*p* < 0.05). This result indicated that Cur-SHNGs might be more beneficial to keep the drug in the skin layer and reduce the further penetration of Cur into the systemic circulation. Therefore, Cur-SHNGs would be more effective in targeting local skin sites than Cur-HNGs.

The amounts of Cur retained in the skin after applying Cur solution, Cur-HNGs and Cur-SHNGs were shown in Figure 5B. After 24 h of transdermal penetration, the amount of Cur retained in skin were 0.12 ± 0.01, 0.34 ± 0.08, and 0.66 ± 0.17 μg/cm^2^ for Cur solution, Cur-HNGs and Cur-SHNGs, respectively. It was obvious that the amount of Cur retained in skin for Cur-HNGs and Cur-SHNGs was 2.83 and 5.50 times higher than that of Cur solution, respectively. It is beneficial when Cur retention in the skin is needed in some local skin diseases such as dermatitis, psoriasis, and burn pain. The significant increase in skin retention of Cur loaded nanogels might be attributed to: 1) The HA molecule has a strong affinity for keratin in the skin and the viscoelasticity of the HA molecule chain allows the formulation to remain in the skin for a longer time (Cilurzo et al., 2014). 2) The nano-sized nanogels is easy to penetrate into the deep layer of the skin through the stratum corneum, and forms a drug depot in the skin to exert a slow-release effect, which is conducive to the accumulation of drugs in the skin tissue to achieve better treatment of local skin diseases (Faisal et al., 2018). 3) Negatively charged drug-loaded nanoparticles could interact with negatively charged skin membranes to increase the transdermal flux of drugs, which in turn could improve drug accumulation in the skin layer (Maione-Silva et al., 2019). Cur-SHNGs showed a significantly higher (*p* < 0.05) drug deposition in skin than that of Cur-HNGs, this might be due to that the helical silk peptide in SOHA chains has special ultrastructure and multi-level structure, which makes Cur-SHNGs have good skin affinity and biological adhesives properties (Dong et al., 2015; Mao et al., 2017). In conclusion, we could say that the increased retention of Cur-SHNGs in the skin is mainly due to the characteristics of the carrier material and the formulation in which the drug is dispersed.

### 3.5 Hematoxylin and eosin staining

**FIGURE 6:**
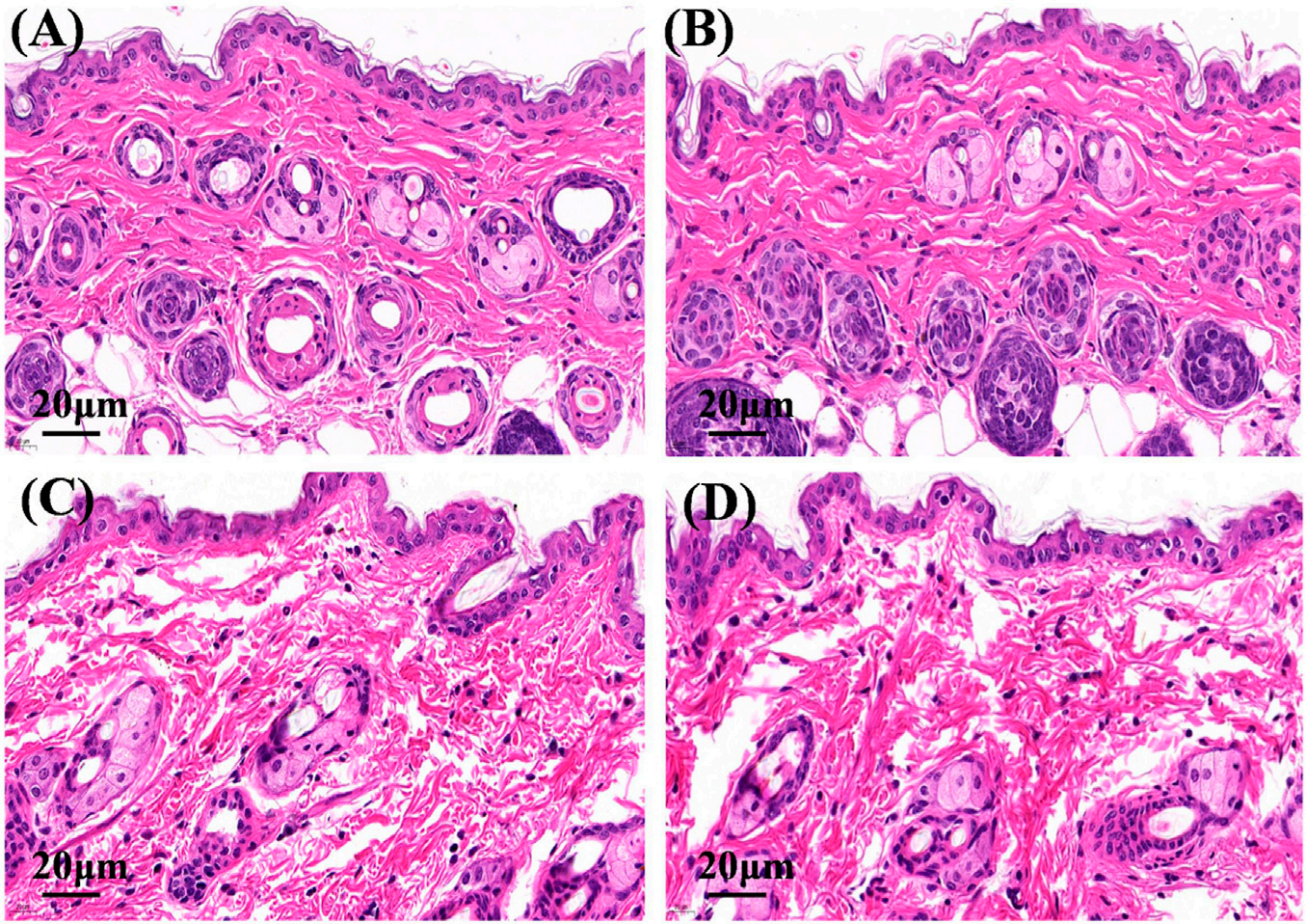
Micrographs of hematoxylin and eosin stained mice skin sections after the mice were treated with physiological saline **(A)**, Cur solution **(B)**, Cur-HNGs **(C)**, and Cur-SHNGs **(D)** (*n* = 3).

On hematoxylin and eosin staining analysis, the nucleus and cytoplasm appeared blue and red (Figure 6), respectively. The structure of the skin tissue in the physiological saline group and the Cur solution group was compact and complete, the epidermal cells were closely arranged, and the edge of cells were difficult to distinguish (Figures 6A,B). After administration of Cur-HNGs or Cur-SHNGs, the skin structure was loose, the intercellular spaces and epidermal cracks of the skin increased, and the cell arrangement in the stratum corneum was disordered (Figures 6C,D). Taken together, Cur-HNGs and Cur-SHNGs altered the skin microstructure, increased skin permeability, and improved transdermal drug delivery, which might be related to the reduced skin density due to the intense skin hydration of HA (Taieb et al., 2012; Taweechat et al., 2020).

### 3.6 Fluorescence imaging

**FIGURE 7:**
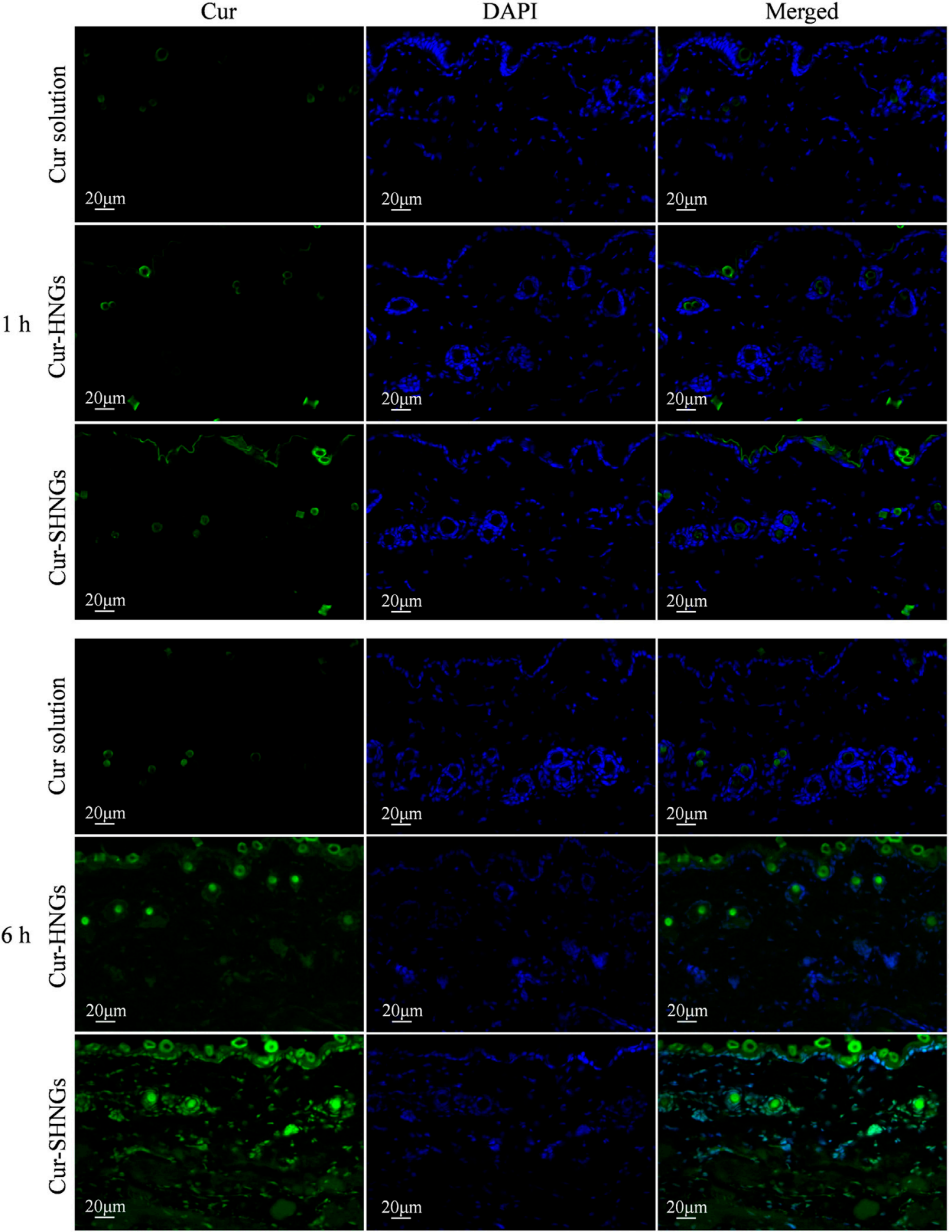
Fluorescence images of longitudinal sections of the skin incubated with Cur solution, Cur-HNGs and Cur-SHNGs at 1 and 6 h (*n* = 3).

In order to effectively improve the topical efficacy of Cur, it is necessary to improve the penetration of the drug into the deep layers of the skin after administration of the formulation. Taking advantage of the characteristic of spontaneous green fluorescence of Cur, *in vivo* skin penetration studies were performed using fluorescence images to confirm the skin permeation and retention enhancement effect of developed formulation, as well as to study the distribution of formulations in the skin. Fluorescence images of mouse skin after treatment with Cur solution, Cur-HNGs and Cur-SHNGs for 1 and 6 h were shown in Figure 7. After 1 h of permeation, only showed weak fluorescence in hair follicles was observed in the skin treated with Cur solution, while in Cur-HNGs group and Cur-SHNGs group, fluorescence was observed in both the upper epidermis and hair follicles, and the fluorescence intensity was stronger than Cur solution. In the case of Cur loaded nanogels, the enhancement of skin fluorescence intensity indicated that they could effectively penetrate the stratum corneum and deliver the drug to the deeper layers of the skin. The fluorescence intensity of all formulations in the skin increased with the extension of permeation time. After 6 h of penetration, the Cur solution group was still mainly distributed in the hair follicles, indicating that it did not penetrate the stratum corneum into the deep layer of the skin, which might be due to the poor permeability of Cur molecules (Yu et al., 2021a), and the hair follicle pathway might be the main pathway for the percutaneous penetration of free Cur. The Cur-HNGs group and the Cur-SHNGs group were distributed in the epidermis, dermis and hair follicles, and showed stronger fluorescence intensity compared with the Cur solution group, indicating that the transdermal penetration ability of the nanogels formulation was much better than that of the Cur solution. The fluorescence intensity of the skin samples from the Cur-SHNGs group was significantly higher than that of Cur-HNGs group, indicating that more drugs were retained in the skin tissue after percutaneous administration of Cur-SHNGs, which might be due to the skin affinity and biological adhesives properties (Yang et al., 2020), these phenomena further confirmed the favorable performance of Cur-SHNGs for transdermal drug delivery.

### 3.7 Assessment of analgesic activity

**FIGURE 8:**
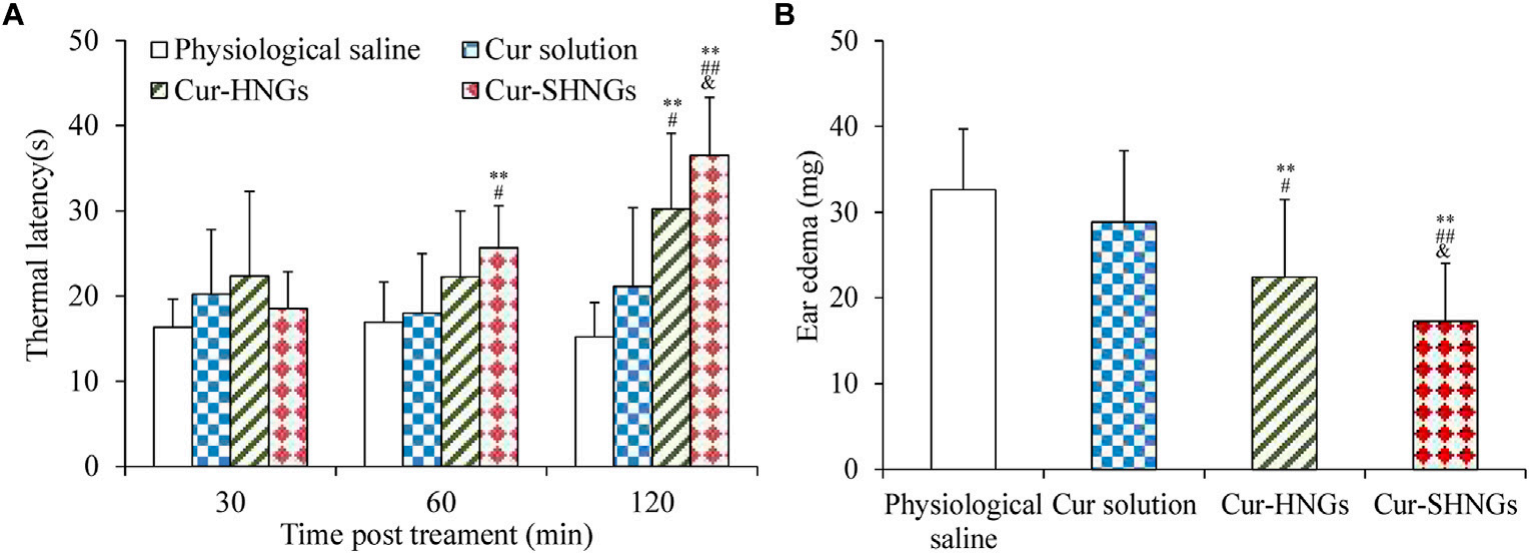
**(A)**Thermal latency of physiological saline, Cur solution, Cur-HNGs, and Cur-SHNGs in mice at various time intervals on the hot plate; **(B)** Effect of physiological saline, Cur solution, Cur-HNGs, and Cur-SHNGs on the weight of dimethyl benzene induced mice ear edema. (**p < 0.01 as compared to physiological saline group, ^#^ *p* < 0.05 and ^##^ *p* < 0.01 as compared to Cur solution group, ^&^ *p* < 0.05 as compared to Cur-HNGs group).

The results of analgesic effects were shown in Figure 8A. From the obtained results, we noticed that the developed Cur-SHNGs formulation exhibited significant analgesic activity at both the 60 and 120 min time points (*p* < 0.05), compared with the physiological saline group and the Cur solution group. At 120 min following topical application of the formulation, the Cur-SHNGs group showed significant analgesic activity compared to the Cur-HNGs group (*p* < 0.05). These results suggested that the Cur-SHNGs treated group had a significantly higher anti-nociceptive effect, which might be due to the fact that Cur-SHNGs improved the penetration of Cur through the stratum corneum and enhanced intradermal retention.

### 3.8 Assessment of anti-inflammatory activity

As can be seen from Figure 8B, the ear edema of the Cur-SHNGs group was 17.26 ± 6.7 mg, while the ear edema of the Cur solution group and the Cur-HNGs group were 28.12 ± 8.58 and 22.43 ± 9.05 mg, respectively. Compared with the Cur solution group and Cur-HNGs group, the ear edema in the Cur-SHNGs group was significantly reduced (*p < 0.05*) when the same dose was topically administered to mice. The ear swelling inhibition rate of Cur-SHNGs was 47.07%, which was 3.41 times and 1.51 times better than that of Cur solution and Cur-HNGs, respectively. These results indicated that Cur-SHNGs had stronger protective and therapeutic effects on dimethyl benzene induced mice ear skin edema when compared with Cur solution and Cur-HNGs. This also further confirmed the advantages of Cur-SHNGs as a transdermal drug delivery system for topical inflammatory diseases.

### 3.9 *In vivo* skin irritation test

**FIGURE 9:**
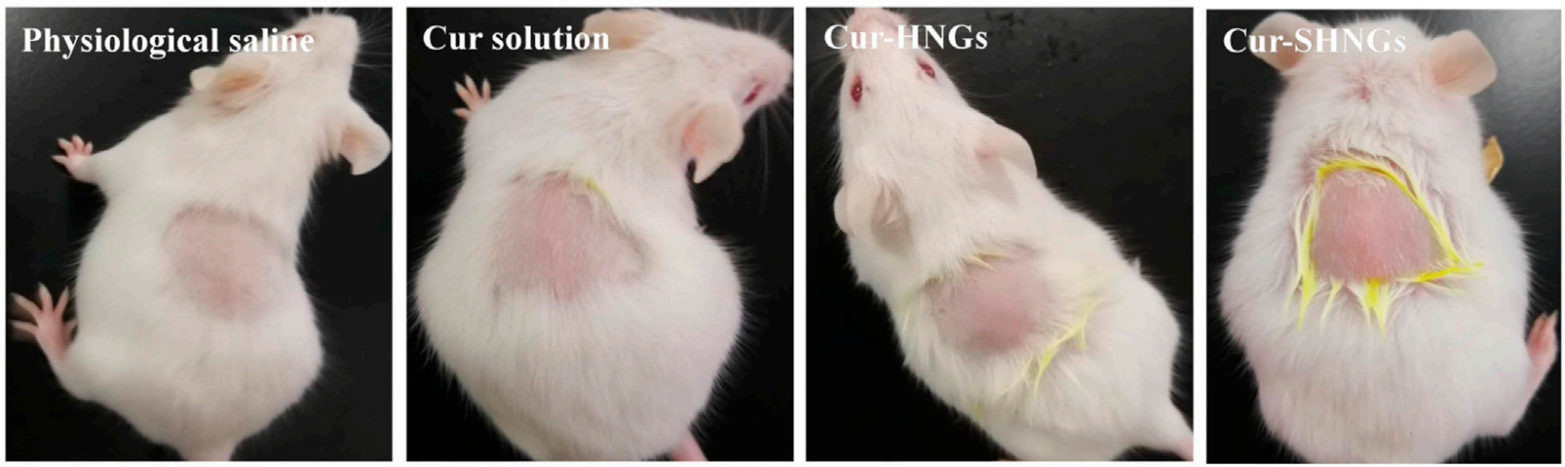
Observation of mice skin appearance at 72 h after exposure to physiological saline, Cur solution, Cur-HNGs, or Cur-SHNGs (*n* = 6).

As shown in Table 1 and Figure 9, all mice tested showed no signs of edema or erythema during the testing period, indicating that the Cur-SHNGs had good biocompatibility and can be safely used as a topical product, which will facilitate improved skin acceptability and patient compliance. In conclusion, despite the Cur-SHNGs exhibited high skin retention of Cur as previously described, no skin irritation was found.

**TABLE 1:** Appearance of mice belonging to the physiological saline (control) group and to the groups treated with Cur solution, Cur-HNGs, and Cur-SHNGs. Experiments were completed on six animals for each group.

Group	Erythema	Edema	Death/total animals
	(Normal for “√”)	(Normal for “√”)	
Control	√	√	0/6
Cur solution	√	√	0/6
Cur-HNGs	√	√	0/6
Cur-SHNGs	√	√	0/6

## 4 Conclusion

In this study, Cur-loaded SOHA nanogels (Cur-SHNGs) were successfully prepared as a novel drug carrier for the enhancement of the topical administration of Cur. The physical properties, *in vitro* release behavior, *in vitro* skin penetration and retention, effects of formulations on skin microstructure, *in vivo* drug activity and biocompatibility of the newly developed nanogels were investigated. *In vitro* skin penetration and retention study revealed higher skin penetration of Cur-SHNGs than that of Cur solution and the highest retention than that of Cur-HNGs and Cur solution. Fluorescence imaging further confirmed the skin permeation and retention enhancement effect of developed Cur-SHNGs. Hematoxylin and eosin staining indicated that nanogels formulation improved transdermal drug delivery by reducing skin density due to the intense skin hydration of HA. In addition, *in vivo* activity tests indicated that Cur-SHNGs had favorable analgesic and anti-inflammatory activity. *In vivo* skin irritation test implied the good biocompatibility of Cur-SHNGs. In conclusion, the Cur-SHNGs should be a promising formulation in the topical drug delivery.

## Data Availability

The original contributions presented in the study are included in the article/supplementary material, further inquiries can be directed to the corresponding authors.
