# Supplementary material for: Silk peptide-hyaluronic acid based nanogels for the enhancement of the topical administration of curcumin
Source: Front Chem. 2022 Sep 19;10:1028372. doi: 10.3389/fchem.2022.1028372 (PMC9527322; doi:10.3389/fchem.2022.1028372)
Supplement: Supplementary file 1 [file Table1.DOCX]

**FIGURE1.** The zeta potential for nanogels of Cur-SHNGs
